# RALB provides critical survival signals downstream of Ras in acute myeloid leukemia

**DOI:** 10.18632/oncotarget.11431

**Published:** 2016-08-20

**Authors:** Craig E. Eckfeldt, Emily J. Pomeroy, Robin D.W. Lee, Katherine S. Hazen, Lindsey A. Lee, Branden S. Moriarity, David A. Largaespada

**Affiliations:** ^1^ Department of Medicine, Division of Hematology, Oncology, and Transplantation, University of Minnesota, Minneapolis, MN 55455, USA; ^2^ Masonic Cancer Center, University of Minnesota, Minneapolis, MN 55455, USA; ^3^ Department of Pediatrics, Division of Hematology and Oncology, University of Minnesota, Minneapolis, MN 55455, USA; ^4^ Department of Genetics, Cell Biology, and Development, University of Minnesota, Minneapolis, MN 55455, USA

**Keywords:** RAS signaling, RALB, TBK1, BCL2, acute myeloid leukemia (AML)

## Abstract

Mutations that activate *RAS* proto-oncogenes and their effectors are common in acute myeloid leukemia (AML); however, efforts to therapeutically target Ras or its effectors have been unsuccessful, and have been hampered by an incomplete understanding of which effectors are required for AML proliferation and survival. We investigated the role of Ras effector pathways in AML using murine and human AML models. Whereas genetic disruption of *NRAS*(*V12*) expression in an *NRAS*(*V12*) and *Mll-AF9*-driven murine AML induced apoptosis of leukemic cells, inhibition of phosphatidylinositol-3-kinase (PI3K) and/or mitogen-activated protein kinase (MAPK) signaling did not reproduce this effect. Conversely, genetic disruption of RALB signaling induced AML cell death and phenocopied the effects of suppressing oncogenic Ras directly – uncovering a novel role for RALB signaling in AML survival. Knockdown of RALB led to decreased phosphorylation of TBK1 and reduced BCL2 expression, providing mechanistic insight into RALB survival signaling in AML. Notably, we found that patient-derived AML blasts have higher levels of RALB-TBK1 signaling compared to normal blood leukocytes, supporting a pathophysiologic role for RALB signaling for AML patients. Overall, our work provides new insight into the specific roles of Ras effector pathways in AML and has identified RALB signaling as a key survival pathway.

## INTRODUCTION

The *NRAS* and *KRAS* proto-oncogenes and genes that regulate Ras activation (i.e. *PTPN11* and *NF1*) are frequently mutated in AML. Moreover, many common mutations in AML occur upstream of Ras (i.e. *FLT3 ITD* and *KIT*), and likely rely on Ras signaling for their oncogenic effects [1–3]. In fact, mitogen-activated protein kinase (MAPK) signaling is activated in more than 80% and phosphatidylinositiol-3-kinase (PI3K) signaling is activated in 50–70% of AML patient samples, suggesting a key role for these Ras effector pathways in AML maintenance [4, 5]. Despite this, there is a limited understanding of how specific Ras effector pathways maintain AML proliferation and survival.

Oncogenic *RAS* mutations are among the most common molecular alterations in human cancer, and thus has been the focus of intense interest for drug development [6]. Ras proteins act as molecular switches to modulate signal transduction by cycling between active guanine triphosphate (GTP)-bound and inactive guanine diphosphate (GDP)-bound states [7]. Ras activation is catalyzed by guanine exchange factors (GEFs) that promote exchange of GDP for GTP in response to growth factor receptor activation, and negatively regulated by the effects of GTPase activating proteins (GAPs) to greatly enhance the inefficient intrinsic Ras GTPase activity [8]. Oncogenic mutations in *RAS* genes, most commonly involving amino acid substitutions at codons 12, 13, and 61 impair GTP hydrolysis, thereby leading to constitutive activation of Ras effector pathways and cellular transformation [9]. Ras-GTP regulates cell proliferation and survival by interacting with a variety of effector enzymes.

The most well characterized transforming pathways downstream of Ras are the MAPK and PI3K effector pathways. Activation of MAPK signaling is initiated by Ras-GTP binding of RAF kinases that results in localization to the plasma membrane and activation of their serine/threonine kinase activity [10, 11]. Activated RAF phosphorylates and activates the mitogen-activated kinase kinases, MEK1 and MEK2, which in turn phosphorylate and activate the mitogen-activated kinases, ERK1 and ERK2. ERK1 and ERK2 phosphorylate a range of proteins including the ETS family transcription factors, JUN, and ultimately drive AP1-mediated cell cycle progression [12]. As is the case for MAPK signaling, Ras promotes PI3K signaling through direct interactions with type I PI3K catalytic subunits leading to membrane localization and kinase activation [13]. Type I PI3Ks subsequently phosphorylate phosphatidylinositol-4,5-bisphosphate (PtdIns(4,5)P_2_) to produce phosphatidylinositol-3,4,5-trisphosphate (PtdIns(3,4,5)P_3_). PtdIns(3,4,5)P_3_ acts as a second messenger activating AKT-dependent and AKT-independent signaling pathways that regulate diverse cellular processes including cell proliferation, survival, motility, and metabolism [14].

The failure to develop effective pharmacologic inhibitors of Ras oncoproteins has led many to conclude that Ras is unruggable [15]. The inherent picomolar affinity of Ras proteins for GTP has precluded the development of effective GTP-competitive direct Ras inhibitors. Alternative approaches to target Ras oncoproteins have involved either 1) disrupting post-translational processing of Ras or 2) inhibiting downstream Ras effector pathways [16]. Clinical studies of farnasyltransferase inhibitors (FTIs) have yielded disappointing results due to alternative biochemical pathways for modifying the carboxy terminal of Ras [8]. Efforts to target Ras effector pathways in myeloid malignancies have primarily focused on targeting the major oncogenic pathways downstream of Ras, MAPK and/or PI3K. Pharmacologic inhibition of these pathways in a variety of AML models has resulted in predominately cytostatic effects [17–20]. Little is known about the role of less-well characterized Ras effector pathways in AML.

There is mounting evidence that the Ras-like (Ral) proteins are critical mediators of Ras-driven transformation, proliferation, migration, and survival of epithelial cancers [21]. For example, a large-scale synthetic lethal RNAi screen uncovered a critical role for TBK1, a downstream target of RALB, in *KRAS*-driven transformation of epithelial cells [22]. Whether RALB signals downstream of Ras have a role in AML has not been established. Understanding how specific Ras effector pathways maintain AML growth and survival represents a critical step toward the rational development of effective Ras targeted treatment strategies. In this study we interrogated the roles of the major oncogenic signaling pathways downstream of Ras – PI3K, MAPK, and RALB – in AML using a tetracycline-repressible *NRAS(V12)* and *Mll-AF9*-driven mouse model (tNM AML) [23], human AML cell lines, and primary patient samples.

## RESULTS

### Suppression of *NRAS(V12)* expression induces apoptosis but not cell cycle arrest in an *NRAS(V12)* and Mll-AF9-driven mouse model of AML

**Figure 1:**
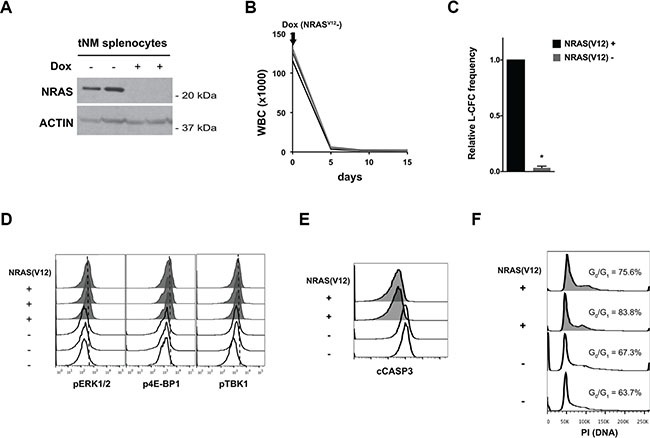
Suppression of *NRAS(V12)* leads to cell death but not cell cycle arrest in *NRAS(V12)* & *Mll-AF9*-driven murine AML cells (**A**) Western blot for NRAS protein levels in splenocytes harvested from tetracycline-repressible *NRAS(V12)* and *Mll-AF9*-driven (tNM) leukemic mice 72 hours after doxycycline (Dox) treatment. (**B**) White blood cell (WBC) counts of SCID Beige mice with tNM AML after initiation of Dox treatment (*n* = 5 mice). (**C**) tNM AML colony formation (L-CFC) from splenocytes harvested tNM leukemic mice after 48 hours of *ex vivo* Dox treatment (error bars = standard error of the mean, *n* = 3 independent experiments, * *P* < 0.001 using two-tailed *t*-test). (**D**) Phospho-flow cytometry analysis of MAPK signaling through phospho-ERK1/2 (pERK1/2), PI3K signaling through phospho-4E-BP1 (p4E-BP1), and RALB signaling through phospho-TBK1 (pTBK1) in tNM splenocytes harvested from leukemic mice at baseline (NRAS(V12)+, shaded histograms) or after 72 hours of Dox treatment (NRAS(V12)-, open histograms). Flow cytometric analysis of (**E**) apoptosis by cleaved Caspase 3 (cCASP3) levels and (**F**) cell cycle by propidium iodide (PI) staining in tNM splenocytes harvested at baseline (NRAS(V12)+, shaded histograms) or 72 hours after Dox treatment (NRAS(V12)-, open histograms). The proportion of cells in the G_0_/G_1_ peak is noted on the cell cycle histograms. Each flow cytometry histogram represents cells from an individual mouse.

We first evaluated the effects of suppressing *NRAS* oncogene expression in the tNM AML model [23]. Briefly, tNM AML cells conditionally express the *NRAS(V12)* oncogene from a tetracycline-response element (TRE) promoter, and *NRAS(V12)* transcription can be suppressed by administering the tetracycline analog doxycycline (Dox). To confirm the *NRAS(V12)* dependence of tNM AML cells we first transplanted tNM AML cells into SCID Beige recipient mice, and monitored their blood leukocyte counts. After treatment with doxycycline, NRAS protein expression was undetectable in splenocytes from leukemic mice by Western blotting at 72 hours (Figure 1A). Suppressing *NRAS(V12)* expression resulted in normalization of leukocyte counts within five days of doxycycline treatment (Figure 1B). Consistent with the *in vivo* results, there was almost complete suppression of leukemic-colony forming cells (L-CFC) after a 48 hour *ex vivo* treatment of tNM AML cells with doxycycline (Figure 1C).

To evaluate the effects of *NRAS(V12)* suppression on MAPK, PI3K, and RALB signaling pathways downstream of Ras, we measured the levels of phosphorylated ERK1/2 (pERK1/2), 4E-BP1 (p4E-BP1), and TBK1 (pTBK1) in tNM AML cells. Suppression of *NRAS(V12)* expression decreased the levels of pERK1/2, p4E-BP1, and pTBK1 (Figure 1D). To investigate the effects of loss of *NRAS(V12)* expression on cell proliferation and apoptosis we evaluated cell cycle profiles and levels of cleaved Caspase 3 (CASP3) by flow cytometry. Suppression of *NRAS(V12)* expression led to apoptotic cell death but did not block cell cycle progression (Figure 1E, 1F).

### Inhibition of MAPK and/or PI3K signaling blocks cell cycle progression but does not induce apoptosis in tNM AML cells

**Figure 2:**
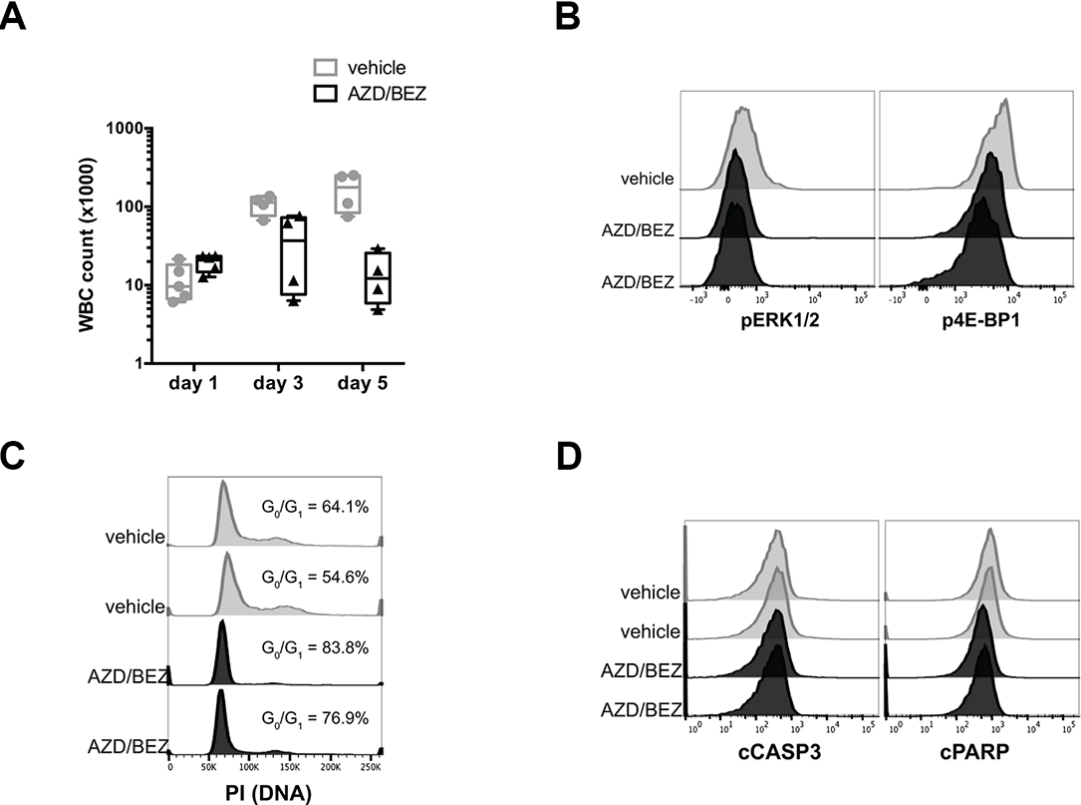
Inhibition of MAPK and PI3K signaling blocks cell cycle progression but does not induce apoptosis in tNM AML cells *in vivo* (**A**) White blood cell (WBC) counts of SCID Beige mice with tNM AML during combination treatment with AZD6244 20 mg/kg twice daily and BEZ235 20 mg/kg daily or control vehicle. Flow cytometric analysis of (**B**) phospho-ERK1/2 (pERK1/2) downstream of MEK and phospho-4E-BP1 (p4E-BP1) downstream of PI3K/mTOR in bone marrow from leukemic mice. Flow cytometric analysis of (**C**) cell cycle by PI staining and (**D**) apoptosis by cCASP3 and cPARP in bone marrow harvested 4 hours after vehicle or drug treatment. Each flow cytometry histogram represents cells from an individual mouse.

To determine whether MAPK and/or PI3K signaling transduce the key survival signals downstream of oncogenic *NRAS* in tNM AML, we pharmacologically inhibited MAPK and PI3K signaling in leukemic mice. Treatment of leukemic mice transplanted with the MEK inhibitor AZD6244 (also known as selumetinib) alone or the PI3K/mTOR inhibitor BEZ235 (also known as dactolisib) alone had no significant anti-leukemic effect *in vivo* despite evidence of inhibition of the target pathways (data not shown). Furthermore, treatment with the combination of AZD6244 and BEZ235 resulted in stabilization of leukocyte counts during the treatment period (Figure 2A) and was not sufficient to reproduce the remission seen after direct suppression of oncogenic *NRAS* expression. We confirmed that the doses of AZD6244 and BEZ235 were sufficient to reduce levels of pERK1/2 and p4E-BP1 (Figure 2B) and block cell cycle progression at the G_0_/G_1_ phase (Figure 2C) in leukemic cells harvested from the bone marrow of treated mice. Notably, combined inhibition of MEK and PI3K/mTOR was not sufficient to reproduce leukemic cell apoptosis as was seen for direct suppression of NRAS oncogene expression (Figure 2D).

**Figure 3:**
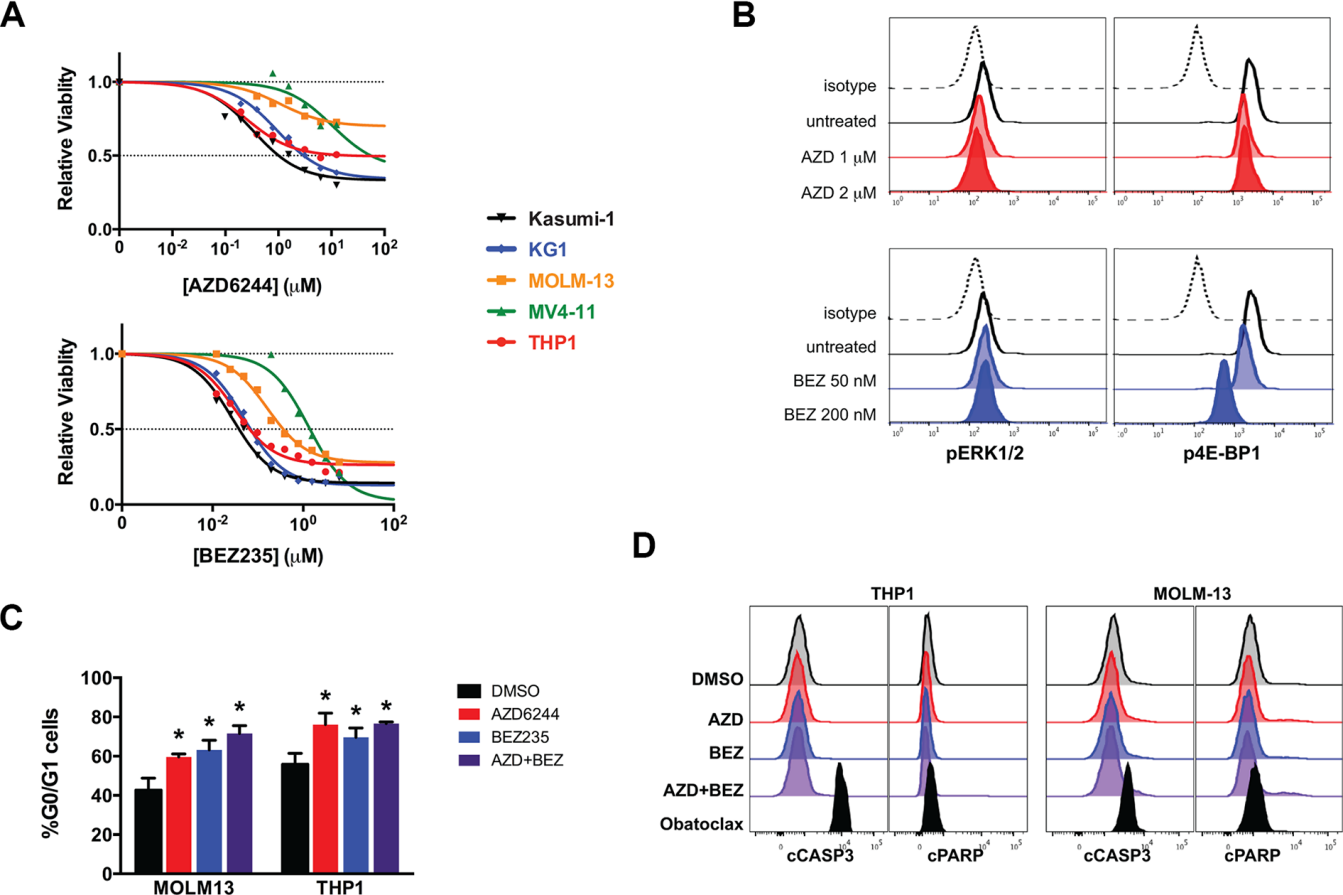
Inhibition of MAPK and PI3K signaling blocks cell cycle progression but does not induce apoptosis in human AML cell lines *in vitro* (**A**) MTS viability analysis of human AML cell lines 72 hours after treatment with AZD6244 (MEK inhibitor) or BEZ235 (PI3K/mTOR inhibitor) (results are the average of 3-5 independent experiments). (**B**) Levels of phospho-ERK1/2 (pERK1/2) downstream of MEK and phospho-4E-BP1 (p4E-BP1) downstream of PI3K/mTOR in THP1 cells 24 hours after treatment by flow cytometry. (**C**) Percentage of cells in the G_0_/G_1_ phase of the cell cycle as determined by propidium iodide (PI) staining using flow cytometry (error bars = standard error of the mean, *n* = 3 independent experiments, * *P* < 0.05 using two-tailed *t*-test). (**D**) Flow cytometric evaluation of cleaved Caspase 3 (cCASP3) and cleaved poly-ADP ribose polymerase (cPARP) levels by flow cytometry of THP1 and MOLM-13 AML cells 24 hours after treatment. Cells were treated with 10 μM AZD6244 alone, 200 nM BEZ235 alone, or the combination of 2 μM AZD6244 and 80 nM BEZ235. Flow cytometry histograms are representative of 3 independent experiments. The BCL2 family inhibitor obatoclax is included in panel (d) as a positive control.

To extend our findings to human AML cells, we evaluated the role of MAPK and PI3K signaling downstream of Ras in *RAS* mutant (THP1) and *RAS* wild-type (KG1, Kasumi-1, MOLM-13, MV4-11) human AML cell lines. In human AML cells, inhibiting MAPK signaling with AZD6244 had a modest effect on viable AML cell numbers (Figure 3A) and suppressed leukemic colony formation (Supplementary Figure S1). Inhibiting PI3K signaling with BEZ235 had a more potent effect on viable cell numbers and leukemic colony formation (Figure 3A and Supplementary Figure S1). There was a dose-dependent reduction in pERK1/2 or p4E-BP1 levels in cells treated with AZD6244 and BEZ235, respectively, with no detectable cross-pathway inhibition (Figure 3B). Targeting MEK and PI3K/mTOR in combination had synergistic effects on the relative viability in three AML cell lines (MOLM13, MV4-11, and THP1), and essentially additive effects in two others (KG1 and Kasumi-1) (Supplementary Figure S2). We next evaluated the effects of AZD6244, BEZ235, or the combination on cell cycle progression and apoptosis in human AML cells. Similar to our findings in tNM AML cells, inhibition of MEK and/or PI3K/mTOR in the human AML cell lines blocked cell cycle progression at the G_0_/G_1_ phase (Figure 3C) but did not induce apoptosis (Figure 3D), and was also not sufficient to reproduce the effects of suppressing *NRAS* oncogene expression in the tNM AML model.

### Genetic disruption of RALB-TBK1 signaling induces apoptosis in leukemia cells

**Figure 4:**
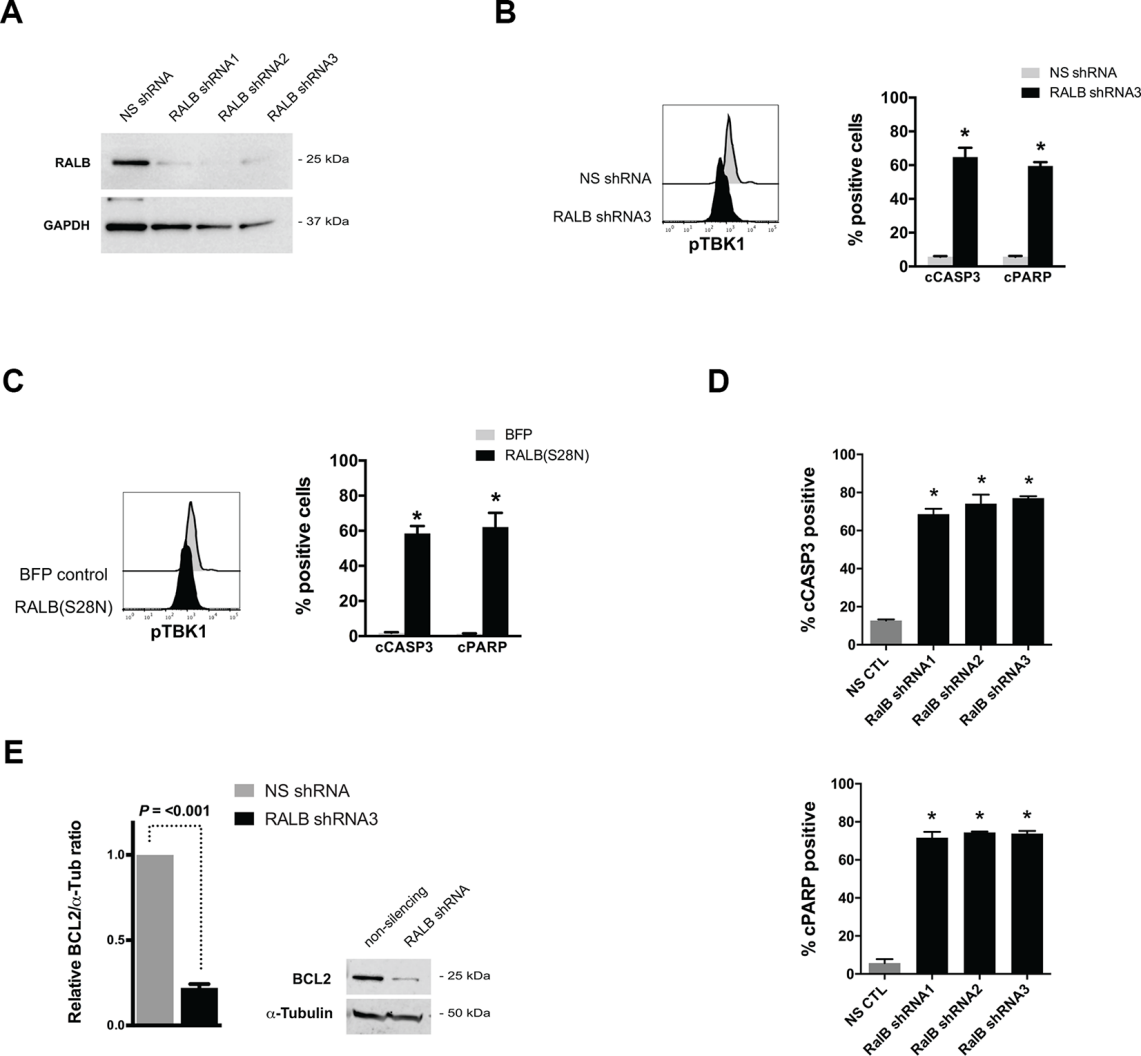
Genetic disruption of RALB-TBK1 signaling induces apoptosis in leukemia cells (**A**) Western blot analysis of RALB and GAPDH protein levels after transfection of K562 leukemia cells with non-silencing (NS) and three independent RALB-targeted shRNAs. (**B**) Representative phospho-TBK1 (pTBK1) levels and percentage of cells with cleaved Caspase 3 (cCASP3) and cleaved poly-ADP ribose polymerase (cPARP) 24 hours after transfection of K562 cells with non-silencing shRNA or RALB-targeted shRNA3 (*n* = 4 independent experiments). (**C**) Representative pTBK1 levels and percentage of cells with cleaved cCASP3 and cPARP 24 hours after transfection of K562 cells with BFP control or dominant-negative RALB(S28N) expression plasmids (*n* = 3 independent experiments). (**D**) Percentage of human THP1 AML cells with cCASP3 and cPARP 48 hours after lentiviral transduction with NS shRNA or RALB-targeted shRNAs (*n* = 3 independent experiments). (**E**) Representative Western blot and relative expression level of BCL2 protein after transfection of K562 leukemia cells with non-silencing (NS) and RALB-targeted shRNA3. Flow cytometry histograms are representative of 3 independent experiments, error bars = standard error of the mean, and *P < 0.05 using two-tailed *t*-test.

The failure of inhibition of MAPK and/or PI3K signaling to recapitulate the effects of suppressing *NRAS* oncogene expression in the tNM AML model led us to hypothesize that RALB, a less well characterized Ras effector pathway, might provide critical survival signals downstream of Ras in AML. To investigate this hypothesis, we genetically disrupted RALB signaling in human leukemia cells. Transfection of K562 cells with RALB-targeted shRNAs led to almost complete knockdown of RALB protein expression, decreased phosphorylation of TBK1, and potently induced leukemic cell death (Figure 4A, 4B). Similarly, enforced expression of a dominant negative form of RALB, RALB(S28N), resulted in decreased phosphorylation of TBK1 and leukemic cell death (Figure 4C). To further validate the role of RALB in AML cell survival and ensure that leukemic cell apoptosis was not due to off-target shRNA affects, we transduced human THP1 AML cells with three independent RALB-targeted shRNAs that each led to potent induction of leukemic cell death (Figure 4D). To provide insight into the mechanism of RALB-mediated cell death, we evaluated the expression of pro-survival BCL2 family genes after knockdown of RALB. RALB knockdown in K562 cells resulted in significant reduction of BCL2 protein expression (Figure 4E) but had no consistent effect on BCLxL or MCL1 expression (data not shown).

### Patient-derived AML blasts have increased levels of RALB-TBK1 signaling compared to normal leukocytes

**Figure 5:**
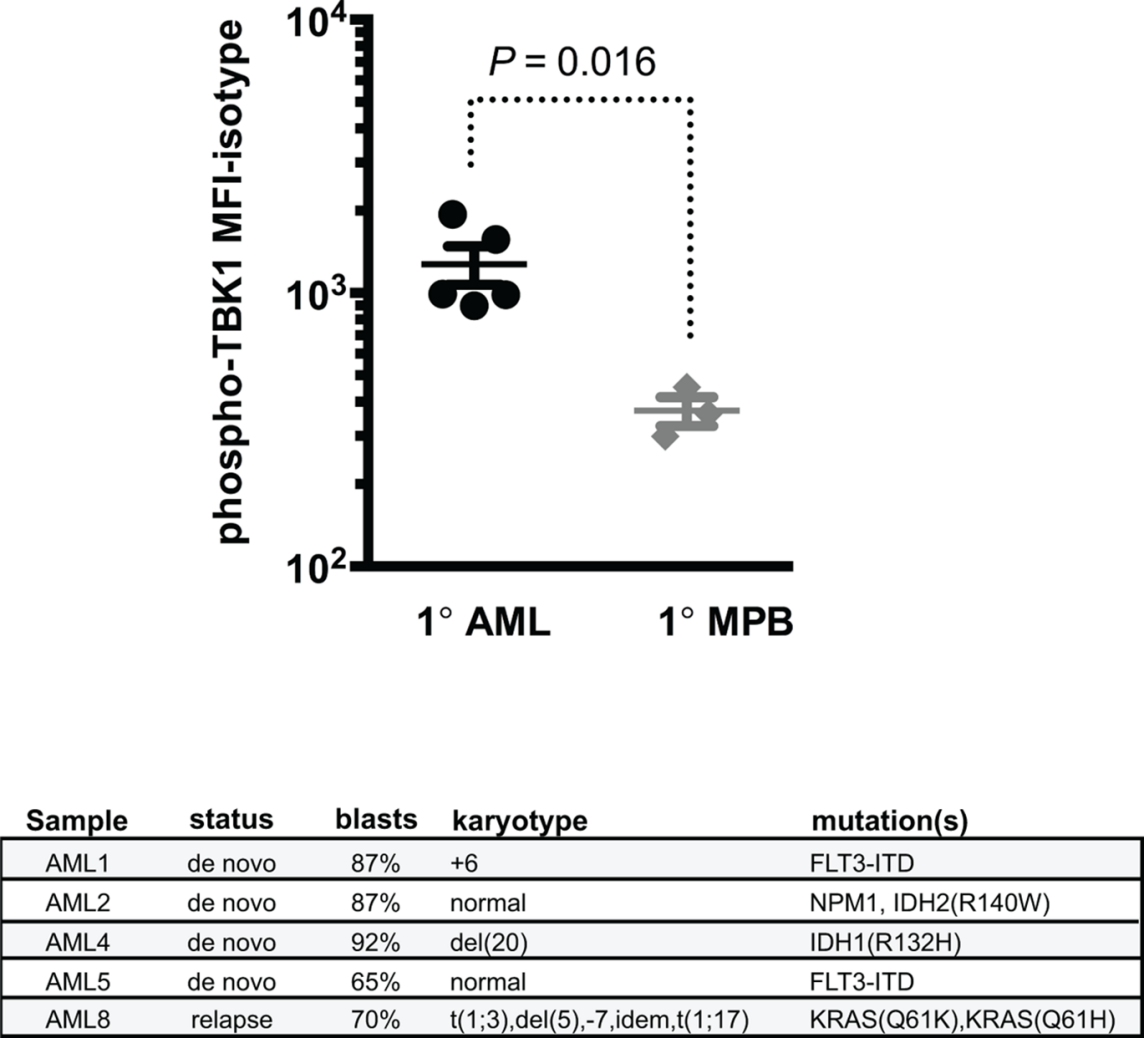
Patient-derived AML blasts have increased levels of RALB-TBK1 signaling compared to normal leukocytes (top) Mean fluorescence intensity of phosphorylated TBK1 (pTBK1) in primary AML patient samples (1° AML, *n* = 5) compared to mobilized peripheral blood mononuclear cells from healthy donors (1° MPB, *n* = 3) by flow cytometry (error bars = mean +/− standard deviation) and (bottom) clinical and genetic characteristics of the primary AML patient samples used.

To establish clinical relevance for our findings we evaluated the activity of RALB-TBK1 signaling in primary patient-derived AML blasts, and found that primary leukemic blasts had higher levels of phosphorylated TBK1 compared to normal blood leukocytes from G-CSF mobilized peripheral blood donors (Figure 5). Notably, increased levels of RALB-TBK1 signaling were seen regardless of cytogenetic/molecular subtype or *RAS* mutation status.

## DISCUSSION

Many common mutations in AML lead to activation of Ras signaling, making Ras and its effectors an attractive therapeutic target. A major obstacle to Ras-targeted therapy for AML is the inherent difficulty in targeting Ras directly. Although targeting Ras effector pathways is a more tenable approach, the optimal targets downstream of Ras for AML are not known. The effects of genetic disruption of *NRAS(V12)* in the tNM AML model supports a critical role of Ras signaling to support AML survival. Surprisingly, inhibition of the major transforming pathways downstream of Ras, MAPK and PI3K, alone or in combination did not reproduce the pro-apoptotic effects of disruption of oncogenic Ras in this model or in human AML cells. While incomplete or transient inhibition of the target pathways could contribute to the differences observed, these findings are consistent with previous work in a variety of AML models [17–20]. Furthermore, single agent MEK inhibition with AZD6244 had only modest and transient activity against relapsed/refractory AML in a recent phase II clinical trial [24]. Targeting aberrant PI3K signaling in AML has also been reported to have primarily cytostatic effects in AML [19]. Several recent clinical trials have reported encouraging results for targeting PI3K in a variety of lymphoid malignancies [25, 26], but the clinical activity of targeting PI3K signaling in myeloid malignancies has not been established. Although feedback between these pathways has been proposed as a mechanism for resistance to MEK- or PI3K-targeted treatment strategies [27], combined targeting of MAPK and PI3K was still insufficient to induce AML cell death. Overall, these data suggests that MAPK and PI3K pathways drive AML proliferation but may be dispensable for AML survival, and led us to hypothesize that alternate Ras effectors transduced critical survival signals for AML cells.

Based on the failure of inhibiting MAPK and/or PI3K signaling to reproduce the effects of direct suppression of *NRAS* oncogene expression on AML cells, we hypothesized that RALB signaling might be a key survival pathway. Murine knockout studies have shown an essential role for either RALA or RALB in *KRAS* driven non-small cell lung cancer cell proliferation [28]. Further evidence for a critical role for RALB-TBK1 signaling downstream of Ras comes from a large-scale synthetic lethal RNAi screen that established a critical role for the RALB-TBK1 signaling in *KRAS*-driven transformation of epithelial cells through it's effects on BCL2 family protein expression [22]. Consistent with this, genetic disruption of RALB signaling led to reduction in BCL2 protein expression, induction of apoptosis, and phenocopied the effects of loss of oncogenic Ras in leukemic cells – supporting a major role for RALB downstream of Ras in AML. The enhanced activation of RALB-TBK1 signaling in primary human AML patient samples compared to normal blood leukocytes underscores the translational potential for these findings. The development of small molecules that directly inhibit Ral function will provide tools to further investigate the therapeutic potential of RAL-based treatment approaches as they become available [29]. Further investigation of pro-survival mechanisms of RALB signaling may provide additional insight into key AML survival pathways and identify other rationale therapeutic targets.

Overall, our results provide new insight into the effects of specific Ras signaling pathways on AML proliferation and survival, and have uncovered a novel role for RALB signaling in AML survival. This work not only refines our understanding of how specific Ras effectors promote proliferation (PI3K and MAPK) and survival (RALB) of AML cells, but also provides a biologic basis to guide the rational development of Ras-targeted treatment approaches for AML patients.

## MATERIALS AND METHODS

### Western blotting

Protein lysates were run on 10% PAGE gels and transferred to a PVDF using the NuPAGE and iBlot systems (Life Technologies). Blots were blocked and stained according to antibody manufacturer's recommendations. Blots were developed using the SuperSignal West Pico ECL kit (Thermo Fisher) and visualized and signals were quantified using the LI-COR imaging system (LI-COR Biosciences). Actin goat polyclonal (sc-1616), NRAS mouse monoclonal (sc-31), and HRP-conjugated secondary antibodies were purchased from Santa Cruz Biotechnology; RALB rabbit polyclonal (3523), α-Tubulin rabbit monoclonal (2144), and GAPDH rabbit monoclonal (2118) antibodies were purchased from Cell Signaling Technologies; and BCL2 mouse monoclonal (610538) antibody was purchased from BD Biosciences.

### Flow cytometry

For staining of intracellular antigens, cells were fixed with 2% paraformaldehyde (Electron Microscopy Sciences) and permeabilized with 90% methanol (Sigma). Cells were stained according to manufacturer's recommendations. Cleaved caspase 3 (Asp175, clone D3E9) PE, cleaved PARP (Aps214, clone 5A1E) Alexa Fluor 647, phospho-4E-BP1 (Thr37/46, clone 236B4) Alexa Fluor 647, phospho-AKT (Ser473, clone D9E) Alexa Fluor 488, phospho-p44/p42 MAPK (Erk1/2, Thr202/Tyr204, clone D13.14.4E) PE, and phospho-TBK1 (Ser172, clone D52C2) PE were purchased from Cell Signaling Technologies; and phospho-TBK1 (pS172, clone J133-587) Alexa Fluor 488 was purchased from BD Biosciences. Cell cycle analysis was performed using the PI/RNase Staining Buffer (BD Pharmingen). Cells were analyzed on an LSR II or Fortessa digital flow cytometer (BD Biosciences), and cell sorting was performed on a FACS Aria II (BD Biociences) at the University of Minnesota Flow Cytometry Resource.

### Cell culture, transfection, transduction, and primary AML samples

Cell lines were originally obtained from ATCC or DSMZ, maintained under standard cell culture conditions, and authenticated by STR analysis at the University of Arizona Genomics Core. RALB-targeted (“RALB shRNA1” V3LHS_396461, “RALB shRNA2” V3LHS_396462, “RALB shRNA3” V3LHS_396465) and non-silencing (NS) control GIPZ shRNA plasmids were obtained from Dharmacon. RALB(S28N) and BFP control expression vectors were generated with the Gateway cloning system (Invitrogen) using a previously described backbone [30], by replacing eGFP with eBFP2 and luciferase with RALB(S28N) from the pBabe puro RalB S28N plasmid (from Channing Der via Addgene #19722). Plasmids were transiently transfected into K562 cells with standard conditions and settings using the Neon Transfection System (Thermo Fisher). VSV-G pseudotyped lentivirus was produced by co-transfecting a 1:2:3 ratio of pMD2.G, pCMVDR8.2 (both from Dider Trono via Addgene #12259 and #8455), and lentiviral expression vector into HEK293 cells using X-treme Gene HP (Roche, Basel, Switzerland). Viral supernatant was harvested after 48 hours, filtered, and used for transduction. Target cells were transduced by co-culture with viral supernatant and 5 μg/mL polybrene overnight, washed, and then cultured for 48–72 hours to allow for expression prior to analysis or sorting. De-identified normal blood mononuclear cells were obtained from G-CSF mobilized peripheral blood (MPB) donors and AML patient samples were obtained from the U of M Leukemia Tissue Bank according to protocols approved by the U of M Institutional Review Board.

### Leukemia colony forming cell (L-CFC) assay

tNM AML cells were cultured in RPMI-1640 medium (Lonza) with 10% fetal bovine serum (Atlas Biologicals) with or without 1 μg/mL doxycycline (Sigma) for 48 hours and then plated in IMDM medium (Lonza) with 30% fetal bovine serum (Atlas Biologicals), 1.275% methylcellulose (R&D Systems), and 2 ng/mL murine GM-CSF (R&D Systems). Human cell lines were treated with pathway inhibitors for 24 hours in standard culture medium and then harvested and plated in MethoCult H4034 Optimum CFC Medium (Stem Cell Technologies) according to manufacturer's recommendations. Colonies were scored after 7–14 days on an inverted microscope.

### Viability cell enumeration

Relative viable cell numbers were determined using the CellTiter 96^®^ Aqueous Non-Radioactive Cell Proliferation Assay (Promega) according to the manufacturer's recommendations. Pathway inhibitors stocks were prepared in dimethyl sulfoxide (DMSO, Sigma), and diluted in cell growth medium to their final concentrations. Half-maximal inhibitory concentrations (IC_50_) and combination indices were calculated using CalcuSyn 2.0 (BioSoft). Inhibitor combinations were considered synergistic if CI < 0.8, additive if CI 0.8–1.2, or antagonistic if CI > 1.2.

### Leukemic mouse models and treatment

All animal studies were approved by the U of M Institutional Animal Care and Use Committee. Briefly, 2 × 10^6^ tNM AML cells were injected via tail vein into 6–10 week old SCID Beige (Charles River) recipient mice without any preconditioning or FVB × C57BL/6 F1 (Jackson Labs) recipient mice after 500 cGy irradiation. Peripheral blood was obtained by retro-orbital blood sampling, and white blood cell (WBC) counts were monitored using a Hemavet 950 (Drew Scientific). Doxycycline treated mice were given a single intraperitoneal dose of 4 mg followed by 5 mg/mL in the drinking water. AZD6244 and BEZ235 (Selleck Chemicals) were diluted in 0.5% hydroxypropylmethylcellulose and 0.1% Tween 80 and administered by oral gavage.

## SUPPLEMENTARY MATERIALS FIGURES


