# Supplementary material for: RALB provides critical survival signals downstream of Ras in acute myeloid leukemia
Source: Oncotarget. 2016 Aug 20;7(40):65147–56. doi: 10.18632/oncotarget.11431 (PMC5323144; doi:10.18632/oncotarget.11431)
Supplement: Supplementary file 1 [file oncotarget-07-65147-s001.pdf]

RALB provides critical survival signals downstream of Ras in acute myeloid leukemia

Supplementary Materials

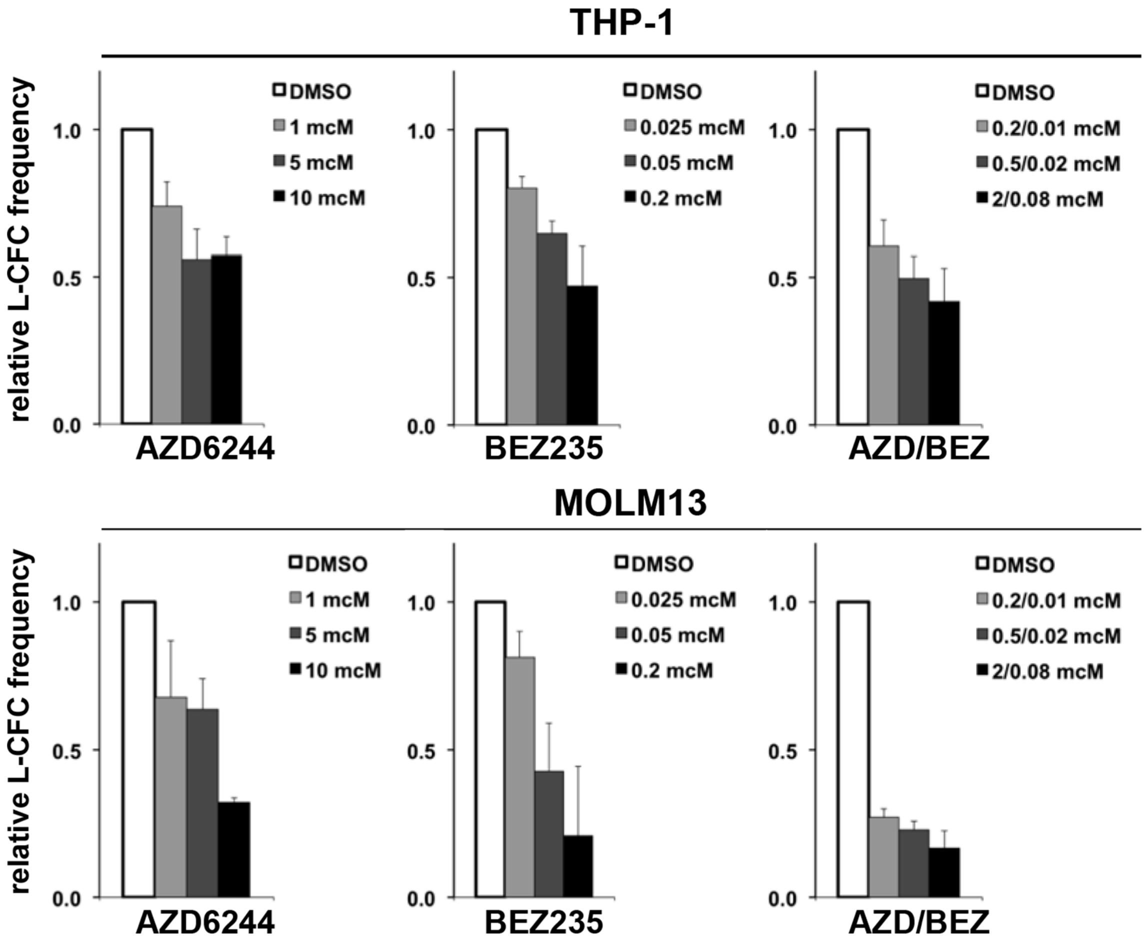

Supplementary Figure S1: Effects of combined inhibition of MAPK and PI3K pathways on AML cell leukemic colony formation. Leukemic-colony forming cell (L-CFC) analysis of AML cells 24 hours after treatment with AZD6244 alone or in combination with a fixed ratio of BEZ235 (*n* = 3–5 experiments, error bars = 1 standard deviation).

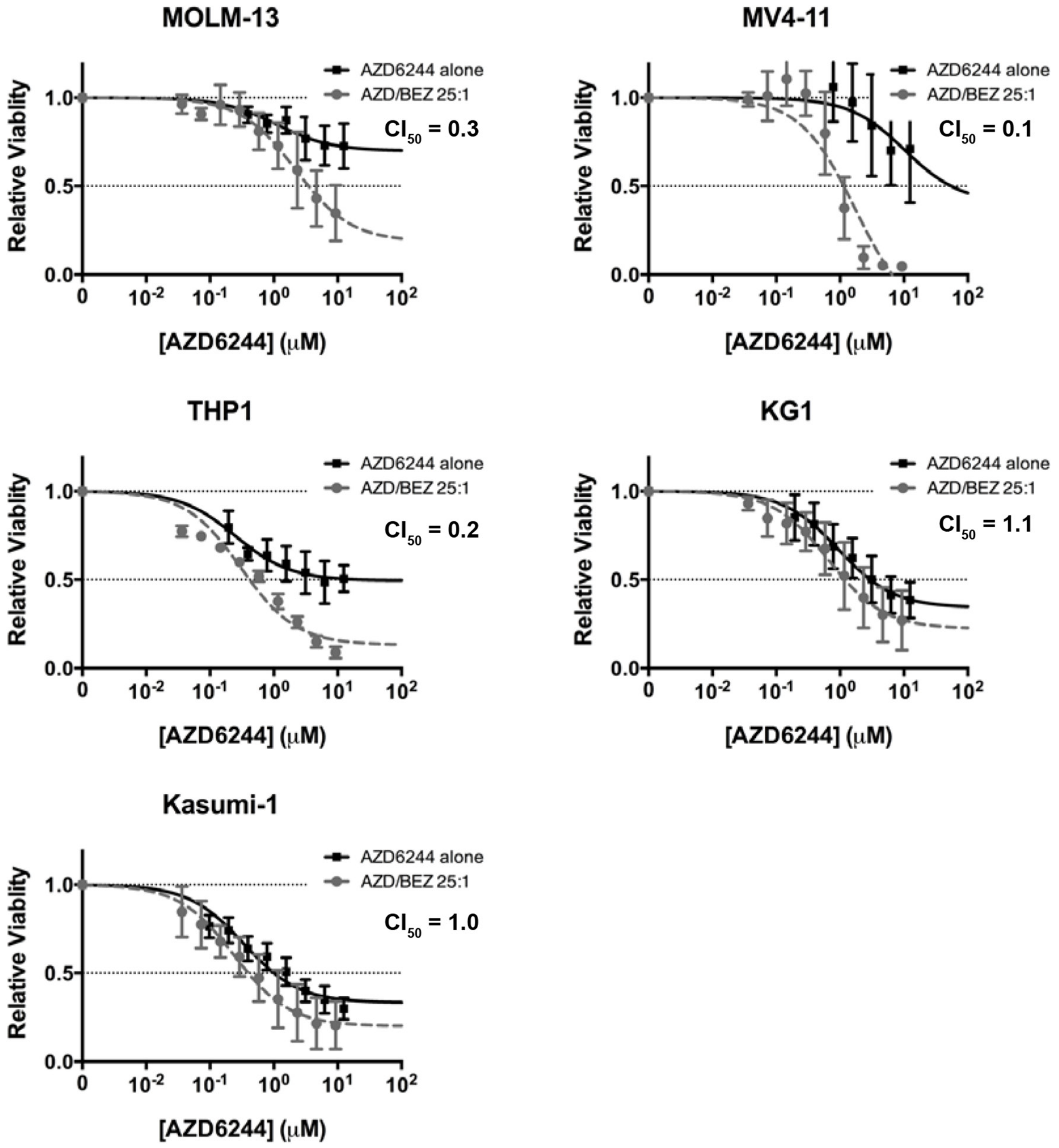

Supplementary Figure S2: Effects of combined inhibition of MAPK and PI3K pathways on AML cell viability. MTS viability analysis of AML cells 72 hours after treatment with varying doses of AZD6244 alone or in combination with a fixed 25:1 molar ratio of BEZ235 (n = 3–5 experiments, error bars = 1 standard deviation, CI_{50} = combination index at 50% effect).
